# Catarrhal Fever

**Published:** 1885-03

**Authors:** K. P. Moore

**Affiliations:** Macon, Ga.


### CATARRHAL FEVER.

READ BEFORE THE MACON MEDICAL SOCIETY, JAN. 20, 1885,
BY K. P. MOORE, M. D., MACON, GA.

The term catarrhal fever is claimed by many to be a misnomer; but to me it is peculiarly significant and expressive; and although it is comparatively a new name, yet the disease, in its epidemic form, is by no means of recent origin; for it has been known and noted from remote antiquity. Cullen called it "Catarrhus E. Contagio;" and under that head in his Nosology, we find frequent references to recorded accounts of epidemic catarrh. Years ago in France the disorder, when prevailing in epidemic form, was styled "*la grippe.*" The Italian writers, putting the cause for the effect, called it influenza; which name became naturalized in our language, and for many years epidemics of catarrhal fever, have been called "epidemics of influenza," a name not expressing the real character of the disease at all; and yet I think a better name than "epidemic bronchitis," the one used by Flint and others. In Prof. Flint's description of this disease, while he calls it "epidemic bronchitis," he says: "It differs materially from ordinary acute bronchitis." "The *bronchial* affection," he says, "is not more intense, but commencing usually with coryza, the inflammation is apt to extend to the frontal and maxillary sinuses, to the lachrymal ducts and conjunctiva, and into the eustachian tube. *Frontal* headache is a *prominent* symptom. It is accompanied by *more marked general symptoms* than ordinary bronchitis, viz. chills, febrile movement, lassitude, debility, anorexia, etc. It is apt to end in free perspiration or diarrhœa. The *disturbance of the system is out of proportion to the pulmonary symptoms.* It is a *general* rather than a *local* disease. * * * It is an *essential fever*, not merely a *local* inflammation with symptomatic febrile movement. It is a *peculiar species of fever*, running a brief career with bronchial inflammation as its anatomical characteristic." (Italics mine.)

Why should Prof. Flint say that "bronchial inflammation was its anatomical characteristic," when he had just before said that it was a *general* rather than a *local* disease, and that it was an *essential fever*, and *not* merely a *local* inflammation? If he had said that bronchial inflammation was *one* of its anatomical characteristics, I should accept it that far; for it cannot be denied that mucous membranes wherever found, are liable to be attacked, and that the bronchial mucous membrane is by *no means* the one *most* affected. Gastro-intestinal complications are very frequent, and Prof. Flint has said in the above quotation, that the disease is apt to end in profuse perspiration or *diarrhœa*. It being true that the bronchial mucous membrane is not necessarily more constantly affected than other mucous membranes, and it also being true that this disease is rather more an essential fever than otherwise, it would be more a misnomer to call it bronchitis than catarrhal fever.

The progress of some of these epidemics affords us an interesting field for study. In the winter of 1870 or 71, there swept across this country a great tidal wave of epizootic diseases among the horses. Setting in at the extreme Northeastern borders, it came in almost a visible wave, extending Northwest and Southeast across the whole face of the country. It traveled at the rate of about seventy-five miles in twenty-four hours. In the winter of 1879 or 1880, a similar epidemic visited the United States. Shortly after, and almost simultaneously with both these epidemics among the horses, especially the last, there came an epidemic of measles, mumps and catarrhal fever. The epidemic of mumps during the winter of 1880, was the most extensive I have ever witnessed, the hideous and ill-shaped faces, muffled in various and sundry dressings to be seen upon every street, told many an amusing, as well as painful tale; and not a few cases of orchitis were docketed upon every professional man's books. It is claimed by many authors that epidemics of catarrhal fever are usually accompanied by epidemics of other diseases, more particularly of the exanthematus type; and these accompanying epidemics of measles and mumps referred to above form curious and interesting problems of study. While it is true that we have not recently had a general *epidemic* of these catarrhal disturbances brought to us in such visible waves, yet the fact remains that we do have more or less catarrhal fever in decided installments every winter; and we naturally look around us for some explanation of it.

Do meteorological changes influence it? Is there within the field of the microscope some bacteria, bacillus, or micrococci to which it can be chargeable? Does mother Earth furnish some genera or contagia which requires a low temperature to develop? Is there any local sanitary evil to which these troubles can be traceable? These are some of the questions which naturally suggest themselves to our minds when we find, within a few days, quite a number stricken down with this peculiar fever all over the community in which we live.

Close observation has convinced me of the contagious or infectious character of the disease, and formerly I believed that it was not dependent upon deficient drainage or other local sources of sanitary evil, but latterly, I am not so sanguine upon this point.

In Bristow's late work on practice, he says: "It is one of the most mysterious, and at the same time one of the most interesting diseases with which we are acquainted. * * * Its origin and diffusion, therefore, have not unnaturally been sought for in some occult telluric, atmospheric, or electrical condition." This author believes it to be infectious in a very high degree.

During the epidemic of 1880, Dr. DaCosta delivered, in the Pennsylvania hospital, a very interesting lecture upon this affection, which was published in the *Medical and Surgical Reporter*. He mentions some of the complications of this disease; chief among which is congestion of the lungs, and its tendency to pass into pneumonia, usually of a severe character. Also its gastro-intestinal symptoms and nervous disturbances. On the question of its mortality Dr. DaCosta says that, from its tendency to nervous depression, and pulmonary complications, this disease has occasioned a great many deaths. My own observation teaches me that few diseases produce greater nervous prostration and weakness, considering the shortness of its duration. Speaking of the nervous depression which usually accompanies an attack of catarrhal fever, I am reminded to speak of another complication which suggested itself to my mind as a probable accompaniment of this disease, and dependent upon the same cause, though happily of not very frequent occurrence. These impressions came to my mind during the prevalence of these catarrhal disturbances of 1871 and 1872, when we had, in addition to the other accompanying diseases before mentioned, also a painful prevalence of cerebro-spinal meningitis. The few cases of meningitis which I saw during that winter were in families and neighborhoods where I had the most serious cases of catarrhal fever, and I came to regard it as a complication of this disease. This idea was then, as now, purely theoretical, but I still think a plausible one.

Perhaps I could not better enumerate the symptoms of this disease than by illustration. On the 24th of last December, I was attacked with rigors and painful chilly sensations; a feeling of weakness and great depression was present, and although I endeavored to keep up and at work, it was done under considerable suffering; and in the early part of the afternoon I was forced to turn in and take my bed. Intense headache, suffusion of eyes, coryza, extremely painful cough, fearful sore throat, and the most intense general soreness and aching were some of the prominent symptoms. By eight o'clock in the evening my stomach became extremely irritable, and free emesis set in. My whole anatomy was now in pain, with much depression, and tendency to congestion of lungs and brain; tongue badly coated and a miserable taste in mouth, and considerable thirst. The next twenty-four hours was anything else than a merry Christmas. About eight o'clock on the evening of the 25th, I was suffering so much my wife sent for my friend, Dr. Fitzgerald, who very kindly came to my relief; and for two or three days he, and my venerable friend and copartner, Dr. Hammond, gave me all necessary attention. My circulation was very much depressed, and indeed did not regain its proper equilibrium for more than two weeks. Although I was able to sit up on the fourth day after my attack, yet it was fifteen or twenty days before I began to feel at all like myself. In detailing my recent attack I have given what I conceive to be a typical case of catarrhal fever, such as we have in more or less abundance every winter, and which is called by so many practitioners, acute bronchitis. That they do not all suffer with all the symptoms which I had, of course goes without saying. Without going into the minutiæ in reference to treatment, I will say that, while it may seem strange that diseases prevailing at such extremes of temperature and season, and dependent, as they must be, upon causes so entirely opposite, should be so amenable to the same course of treatment; and yet it seems true that our great anti-malarial remedy never yielded more satisfactory results in malarial troubles, than in this. This disease is almost always complicated with more or less so-called biliousness, sometimes producing more or less jaundice; hence I almost always begin the treatment with a cathartic dose of calomel. My favorite prescription to begin with is calomel grs. x. pulv. rhubarb grs. v. sulph. quinia grs. v. M. and put into one capsule to be taken at once; to be followed with four grs. of quinine and two of dovers powder every three or four hours. Next in the order of treatment, if indeed it does not rank equal to the quinine, is opiates; pain and restlessness must be held in abeyance, and nerve force cannot be better husbanded than by some form of opium. Dovers powder, considering its diaphoretic qualities, is a good form; but morphia will sometimes have to be resorted to in the beginning. Choral to secure sleep, and whisky to sustain the depressed circulation. In view of the depressed vital force and general prostration, nourishments must not be lost sight of; milk punches, hot or cold as may be most grateful to the stomach, affords the best form, egg-nog the same way, chicken or beef tea, etc.

Turpentine is one of our main remedies, and may be used, as pleasantly suggested by my good friend, Dr. S. H. Gray, "externally, internally, and eternally." Other valuable remedies such as carb. ammonia, camphor, etc., come in to fill certain indications in each individual case. Tr. digitalis may be used with stimulants, to give tone to the circulation. I have mentioned only a general outline of my plan of management, and leave it to the better judgment of every practitioner to meet the special indications of each individual case.

